# The Role of Defense Mechanisms in Self‐Injurious Behaviors: A Systematic Review

**DOI:** 10.1002/jclp.70113

**Published:** 2026-02-21

**Authors:** Ornella Montebarocci, Laura Sirri

**Affiliations:** ^1^ Department of Psychology University of Bologna Bologna Italy

**Keywords:** defense mechanism, non‐suicidal self‐injury, self‐harm, self‐injurious behavior, self‐injury, suicide

## Abstract

**Introduction:**

The identification of psychological risk factors for suicide and self‐harm behaviors is necessary for preventive and therapeutic strategies. The aim of this article is to systematically review research articles concerning the relationship between defense mechanisms and self‐injurious behaviors according to PRISMA criteria.

**Methods:**

PubMed, Web of Science, PsycInfo, and Scopus electronic databases were searched from inception to 01/27/2025 by combining the keywords “defense mechanism*” AND “suicid*” OR “non suicidal” OR “self harm” OR “self injury.” Risk of bias was examined with the Study Quality Assessment Tools of the National Heart, Lung, and Blood Institute.

**Results:**

A total of 428 records was yielded by electronic search and after removal of duplicates and application of inclusion criteria 24 articles fulfilled the inclusion criteria. Six additional articles were added by means of references search, leading to a total of 30 included articles. About half of the studies were performed in adult samples and the remaining in children and adolescents. Most of the articles included patients recruited in psychiatric settings. Both in adults and adolescents, a significant association between immature defenses, especially displacement, splitting, acting out, and projection, and increased risk for self‐injurious behavior was found. Conversely, mature defenses were inversely associated with self‐harm ideation and behaviors.

**Conclusions:**

Defenses represent an area where early intervention could be implemented as part of a preventive strategy to reduce self‐injurious behaviors. A routine evaluation of defensive functioning should be methodically included in the psychological assessment of patients with self‐harming ideation or behaviors.

## 1 Introduction

Suicide is a global health concern since it is one of the major causes of death in all the regions of the world (World Health Organization 2025) and it results in significant psychosocial and economic burdens (Jain et al. 2024). The World Health Organization estimated globally 727,000 deaths by suicide every year and included the reduction of suicide mortality as an indicator of the United Nations Sustainable Development Goals under target 3.4 (World Health Organization 2025). According to a recent meta‐analysis across 71 countries spanning 1900–2021, suicide deaths are underestimated and, after correction for underreporting, they would be more than one million per year (Meda et al. 2025).

In children and adolescents aged 5–19 years, suicide accounts for 4.2% of total deaths (Liu et al. 2022). In people aged 15–29 years, suicide is ranked as the global third cause of death (World Health Organization 2025). According to a US nationally representative cross‐sectional study, the overall annual suicide attempts rate in the general adult population significantly increased from 481.2 to 563.9 per 100,000 adults from 2008 to 2019 (Bommersbach et al. 2022). In the last decade, suicide rates have increased especially among middle‐aged adults (Qin et al. 2022), with suicide mortality having the highest impact on life expectancy in the 55–64 age group (Sagna et al. 2020). In particular, middle‐aged men have the highest suicide rates in several high‐income countries (Graney et al. 2024).

In the context of suicidal behavior, different non‐fatal self‐injurious behaviors have been described, including suicidal ideation, suicidal attempts, and non‐suicidal self‐injury (Griep and MacKinnon 2022; Sánchez‐Teruel et al. 2020; Udupa et al. 2023). Despite different efforts to provide a classification of these self‐injurious phenomena, a consensus on terminology has not been reached (Silverman and De Leo 2016). Categorizing self‐directed violent behaviors as suicidal or non‐suicidal is especially difficult. In particular, the role of intent and lethality in such a categorization remains an object of debate (Silverman and De Leo 2016). De Leo et al. (2021) conducted the International Study of Definitions of English‐Language Terms for Suicidal Behaviors (ISDELTSB) to explore the agreement level about suicide‐related terminology between experts. This study provided the basis for developing a transcultural nomenclature of several self‐injurious behaviors. According to the results of the ISDELTSB, recommended definition of suicide is as follows “an act resulting in death which is initiated and carried out by an individual to the end of the action, with the knowledge of a potentially fatal result, and in which intent may be ambiguous or unclear, may involve the risk of dying, or may not involve explicit intent to die.” Proposed definitions of suicide attempt and suicidal ideation are “an act in which a person harms himself or herself, with the intention to die, and survives” and “to think of suicide with or without suicidal intent, or hope for death by killing oneself, or state suicidal intention without engaging in behavior.” Self‐harm is defined as “a non‐fatal act in which a person harms himself or herself intentionally, with varying motives including the wish to die” (De Leo et al. 2021).

Non‐suicidal self‐injury (NSSI) is another term frequently used in the literature on self‐injurious behaviors. According to the DSM‐5, it is an intentional self‐inflicted damage to the surface of one's own body of a sort likely to induce bleeding, bruising, or pain, with the expectation that the injury will lead to only minor or moderate physical harm (American Psychiatric Association 2013). NSSI involves the absence of suicidal intent (American Psychiatric Association 2013). However, it was found to be a strong predictor of subsequent suicidal attempts (Kiekens et al. 2018; Park et al. 2024).

The identification of risk factors for self‐injurious behaviors is necessary to develop preventive strategies. Some conditions significantly increase the risk for suicide and self‐harm behaviors, including the presence of a psychiatric disorder, severe medical conditions, adverse events, and being imprisoned (Favril et al. 2023; Mundt et al. 2024; Pelizza et al. 2025; Richardson et al. 2024; Zhou et al. 2024). However, the role of psychological factors (e.g., personality traits or coping strategies) in suicide‐related behaviors seems to be underexamined and it needs further investigation. Defense mechanisms are likely to be one of the major determinants of self‐injurious behaviors, since they regulate one's psychological functioning and determine the response to external events. The concept of defense was first introduced in psychoanalysis and later it has been incorporated by other fields of psychology, including cognitive, social, developmental, and personality psychology (Cramer 2015). Cramer (2015), after reviewing empirical studies on defense mechanisms, defined them as cognitive processes that function to protect the individual from excessive anxiety or other negative emotions, loss of self‐esteem and, in the extreme, the loss of self‐integration.

The concept of defense has now reached a stable convergence of clinical and empirical interests, establishing it as a keystone of mental functioning (Di Giuseppe et al. 2019). Moreover, defense mechanisms hold predictive value, overseeing the diagnostic and assessment process, and represent a key area of intervention for preventive actions and the promotion of psychological change (Conversano et al. 2023; Fiorentino et al. 2024).

Several authors propose that defenses are organized according to a hierarchical schema (Vaillant 2000), with defenses situated at a higher or more mature hierarchical level being associated with specific developmental trajectories characteristic of “normal” functioning (Cramer 2015). Such mature defenses allow for a more conscious modulation and expression of desires and needs (Perry 2014). In contrast, defenses at a lower or more immature hierarchical level are associated with “pathological” functioning, as they are characterized by reduced awareness and psychological flexibility (Békés et al. 2023; Perry and Bond 2012).

Among hierarchical models, those proposed by Kernberg and Vaillant are particularly noteworthy. According to Vaillant (2000), defenses are connected to key aspects of mental functioning, playing a crucial role in shaping both our psychopathological profile and overall adaptation. Vaillant places defenses along a continuum reflecting two distinct yet intimately related dimensions: immaturity‐maturity and mental illness‐mental health. Mature defense mechanisms encompass defenses such as sublimation, repression, and anticipation, whereas immature mechanisms include defenses like projection, dissociation, and acting out. Between these two extremes, Vaillant identifies two additional intermediate groups, classified as neurotic and narcissistic.

According to Kernberg (1984), defenses are classified into three levels: the level of mature defenses, which includes mechanisms such as anticipation, altruism, and humor; the level of removal‐based or neurotic defenses, where conflicting themes are banished from consciousness, encompassing repression, affect isolation, intellectualization, reaction formation, and displacement; and finally, the level of splitting‐based defenses, where aspects of conscious experience are separated to avoid conflict, including mechanisms such as splitting, projective identification, omnipotent control, devaluation, denial, and acting out.

In Kernberg's model, mature defenses allow the individual to engage in anxiety‐provoking situations while experiencing minimal emotional distress and without needing to exclude conflictual elements from awareness. Neurotic defenses exclude certain cognitive or emotional aspects of experience. Primitive defenses function to keep idealized and persecutory aspects of reality separate, invariably distorting reality and impoverishing interpersonal relationships through rapid cycles of idealization/devaluation, “emotional storms,” lack of impulse control, and abandonment anxieties.

The study of defenses entails examining how they shape the conditions for the development of psychopathological symptoms, including those that may predispose individuals to engage in suicidal or self‐harm behaviors when coping with situations that exceed the subjective capacity for tolerance. Moreover, defense mechanisms have been identified as intrapsychic correlates of suicidal behaviors in various studies as they help reduce anxiety and protect the self against harmful thoughts or feelings (Lee et al. 2022). Although the critical role defensive configuration plays in preventing, determining or conversely facilitating self‐injurious behaviors, no systematic review of this body of work has been conducted to date.

The aim of this paper is to systematically review research studies examining whether defense mechanisms are significantly associated with measures of self‐injurious behaviors. We included both studies on patients with suicidal attempts or with NSSI and studies where risk for self‐harm behaviors was assessed in clinical and non‐clinical samples.

## 2 Methods

This systematic review was performed according to the Preferred Reporting Items for Systematic Reviews and Meta‐Analysis (PRISMA) guidelines (Moher et al. 2009; Page et al. 2021).

### 2.1 Data Sources and Search Strategy

The PubMed, Web of Science, PsycInfo, and Scopus electronic databases were searched from inception to 01/27/2025. In each database, the following keywords were combined: “defense mechanism*” AND “suicid*” OR “non suicidal” OR “self harm” OR “self injury.” Searches were performed within the Title/Abstract section in PubMed and in the Abstract section in Web of Science, PsycInfo, and Scopus. In PsycInfo, dissertations were excluded. Both in PsycInfo and in Scopus, English language was set as a filter. A manual search of the reference lists of the articles included in the review was also performed.

### 2.2 Selection Criteria and Process

Articles were included in the systematic review if they satisfied the following inclusion criteria: (a) reporting data on the association between defense mechanisms and suicidal or self‐harm ideation or behaviors; (b) being quantitative research articles; (c) being published in English; (d) having used psychometric instruments specifically developed and validated to assess defense mechanisms.

Records were excluded if they: (a) were qualitative studies, reviews, book chapters, case reports, letters, editorials, conference papers, and dissertations; (b) were published in a language different from English; (c) did not assess defense mechanisms by means of validated psychometric instruments.

The two authors independently screened the records and then compared their decision as to the inclusion or exclusion of the articles in the review. In the case of initial disagreement, final agreement about whether to include or not an article in the review was reached after discussion.

### 2.3 Data Extraction and Analysis

All the retrieved records were entered into an electronic form where the following information was extracted: database (PubMed, Web of Science, PsycInfo, or Scopus), keywords, first author, title, year of publication with volume, issue and page numbers, and journal/source. For each record, in the electronic form it was also specified whether it was a duplicate, whether it was excluded by examination of title/abstract (with reason, for excluded records), full text availability for articles not excluded by title/abstract, and inclusion or exclusion after full text examination (with reason, for excluded records).

For included articles, the following information was also extracted: country where participants in the study were recruited, sample characteristics, the psychometric instrument used to assess defense mechanisms, type of self‐injurious behavior examined in the study, and main findings concerning the association between defense mechanisms and self‐injurious behaviors. The electronic form is available in AMS Acta Institutional Research Repository, at https://doi.org/10.6092/unibo/amsacta/8634, URI https://amsacta.unibo.it/id/eprint/8634.

Risk of bias of each included article was assessed through the Study Quality Assessment Tool for observational cohort and cross‐sectional studies of the National Heart, Lung, and Blood Institute (NHLBI). This tool provides an in‐depth examination of the methodological quality of observational cohort and cross‐sectional studies, through 14 questions ranging from clarity of research questions to statistical adjustment for potential confounding variables. It leads to a classification of the articles as good, fair, or poor (National Institutes of Health 2021). Supporting Information S1: Table 1 shows the risk of bias assessment of the included articles in detail.

The electronic searches yielded a total of 428 records: 95 from PubMed, 104 from Web of Science, 89 from PsycInfo, and 140 from Scopus. Two hundred and fifteen duplicates were removed, leaving 213 records to be examined: 164 of them were excluded by title/abstract examination. Reasons for exclusion were as follows: relevance to the topic of the review (*n* = 96), type of publication (*n* = 57), and language (*n* = 11).

**FIGURE 1:**
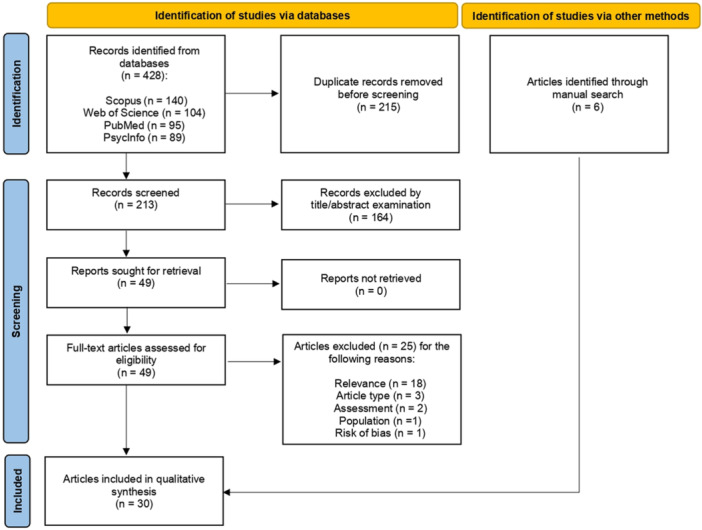
Flow diagram for articles selection process according to PRISMA guidelines.

The full text of the 49 articles not excluded by title/abstract was examined: 25 articles were excluded and 24 were included in the review (see Figure 1). The reason for exclusion of the 25 articles after full text examination was as follows: relevance to the topic of the review (*n* = 18), type of publication (*n* = 3), type of population (*n* = 1), insufficient information about the assessment of defense mechanisms or self‐injurious behavior (*n* = 2), significant potential for bias due to analysis of only the participants who completed the intervention in a randomized controlled study (*n* = 1).

Six additional articles were added by means of references search (Peng et al. 2023, 2024; Pfeffer et al. 1982, 1984, 1986, 1988), leading to a total of 30 included articles.

## 3 Results

### 3.1 Included Articles

Of the total number of articles included in the review, 43.3% were conducted with Asian population, 33.3% were from USA, and 23.3% from European countries.

As to the samples' characteristics, 57% of the 30 included articles describe findings in adult samples, 23% included adolescents, 17% included children, and 3% included a sample consisting of both adolescent and adult outpatients.

Seventy‐seven percent of the articles included patients recruited in psychiatric settings: 78% of these articles included at least one sample with suicidal behavior, and 10% included at least one sample with NSSI. Three percent of the articles were performed on bariatric surgery patients, 3% on patients with alcohol dependence, and 17% in non‐clinical samples: University students with at least one lifetime episode of self‐injury, preadolescents recruited in school settings, non‐clinical adolescents, and imprisoned men.

Defense mechanisms were assessed mainly by means of the 40‐item version of the Defense Style Questionnaire (Andrews et al. 1993), the Defense Mechanisms Inventory (Gleser and Ihilevich 1969), the Life Style Index (Plutchik et al. 1979), and the Ego Defense Scale of the Child Suicide Potential Scales (Pfeffer et al. 1979).

According to the Study Quality Assessment Tools of the National Heart, Lung, and Blood Institute, 50% of the articles were rated as good and 50% as fair (see Supporting Information S1: Table 1).

### 3.2 Association Between Self‐Injurious Behaviors and Defense Mechanisms: Mature and Immature Defenses

Self‐injurious behaviors were significantly associated with higher use of immature defenses (Brody and Carson 2012; Corruble et al. 2003, 2004; Dedic et al. 2010, 2019; Ishfaq and Kamal 2024; Moradinazar et al. 2019; Mozafari et al. 2024; Peng et al. 2023; Xia et al. 2023) and, to a lesser extent, of neurotic defenses (Corruble et al. 2003; Güzel et al. 2022; Moradinazar et al. 2019; Mozafari et al. 2024; Xia et al. 2023). Some studies also found an inverse association between self‐harm ideation and behaviors and mature defenses (Brody and Carson 2012; Moradinazar et al. 2019; Mozafari et al. 2024; Peng et al. 2023; Xia et al. 2023). The association between self‐injurious behaviors and defense mechanisms also appears to be valid in the non‐clinical population (Brody and Carson 2012; Sarno et al. 2010).

Indeed, the data consistently emphasize that immature and neurotic styles contribute to an increased risk for self‐injurious behavior, both in clinical and non‐clinical populations. It may be hypothesized that people using more immature and neurotic styles are more susceptible to die for suicide when facing stressful situations because of more dysfunctional personality traits, including aggression, anger, and victimization, or a history of negative life events (Bakhtiari et al. 2023; Dedic et al. 2019; Lee et al. 2020; Moradinazar et al. 2019; Mozafari et al. 2024). Some studies highlight how anxiety and social dysfunction are moderators of defensive functioning, which in turn influences the inclination for self‐destructive behaviors in adult psychiatric populations (Lee et al. 2022; Mozafari et al. 2024). Studies in imprisoned individuals, obese populations who have undergone bariatric surgery, individuals with alcohol dependence, and those with a history of suicide by poisoning suggest that, when individuals lack adequate resources to cope with perceived demanding situations, they engage in defensive strategies that worsen rather than alleviate their difficulties (Dedic et al. 2010; Greenwald et al. 1994; Güzel et al. 2022; Ishfaq and Kamal 2024).

Some studies addressed the role of defenses in NSSI, where self‐injurious behavior appears to be the pivotal factor influencing the severity and quality of psychopathology. Peng et al. (2023, 2024) specifically found that adolescents with major depressive disorder and NSSI exhibit more immature defenses, more severe depressive symptoms, including suicide attempts, and greater borderline traits compared to adolescents with major depressive disorder without concomitant NSSI. Moreover, compared to non‐clinical adolescents, those with depression show a reduced reliance on mature defensive strategies. Similarly, Xia et al. (2023) reported that psychiatric adolescent inpatients demonstrate fewer mature defenses and a greater reliance on immature defenses compared to controls. These findings are consistent with studies in non‐clinical samples. For instance, Brody and Carson (2012) suggested that both NSSI and deliberate self‐harm are associated with greater use of immature defenses and reduced use of mature defenses in non‐clinical adolescent samples. Sarno et al. (2010) observed that University students with episodic or recurrent self‐injury show more severe clinical symptoms, and a greater use of immature defenses compared to non‐clinical adolescents without self‐injurious behaviors. However, they noted no significant differences in the use of mature defenses, making these adolescents potentially indistinguishable at times, particularly during periods of overall good functioning.

It is reasonable to hypothesize that understanding defensive styles is crucial for assessing the role self‐injurious behavior plays in suicidal attempts, particularly when NSSI is considered a precursor to suicide—two phenomena differing along a continuum of severity (Park et al. 2024). Alternatively, when NSSI is conceptualized as an independent symptom, defined by the absence of suicidal ideation (as its definition suggests), defensive styles may still provide valuable insights into their unique role and implications (Halicka and Kiejna 2018).

According to some studies, the significant association between immature and neurotic styles and suicidal ideation and attempts may be explained by increased impulsivity (Corruble et al. 2003; Mozafari et al. 2024). The role of impulsivity in suicide is often considered to be complex. It has been theorized that rather than a direct relationship, impulsivity has a distal relationship to suicidal behavior by increasing one's exposure to painful and provocative events (Anestis et al. 2014).

### 3.3 Association Between Self‐Injurious Behaviors and Defense Mechanisms: Specific Defenses

As to the specific defenses, in adult samples especially displacement, splitting, acting out, and projection were found to be significantly associated with self‐harm behaviors (Apter et al. 1989b; Corruble et al. 2004; Dedic et al. 2010; Sarno et al. 2010).

In young patients with suicidal behavior, many studies converge in identifying defenses such as introjection, regression, repression, and displacement, and, to a lesser extent, denial as those most commonly observed (Apter et al. 1997; Fennig et al. 2005; Greenwald et al. 1994; Pfeffer et al. 1995). Finally, turning against the self is a defense commonly found in suicide attempters both in adolescent (Borst and Noam 1993; Foto‐Özdemir et al. 2016; Recklitis et al. 1992) and in adult samples (Scholz 1973). Table 1 depicts the main features and findings of the included articles.

**TABLE 1:** Association between defense mechanisms and self‐injurious behaviors.

Authors	Country	Sample	Assessment of defense mechanisms	Outcome measures	Main findings
Apter et al. (1989b)	USA	60 psychiatric inpatients: 50% admitted because of suicide attempts (50% men, mean age 29.8 ± 8.6 years) and 50% for other reasons (50% men, mean age 32.5 ± 10.7 years)	Life Style Index (LSI; Plutchik et al. 1979)	Suicide Risk Scale (Plutchik et al. 1989)	Suicidal inpatients scored higher on regression than non‐suicidal inpatients (*p* < 0.01). Suicide risk was associated with higher scores on repression (*p* < 0.001) and displacement (*p* < 0.01), and with lower scores on denial (*p* < 0.001).
Apter et al. (1997)	Israel	55 suicidal adolescent inpatients (30.9% males, mean age 16 ± 2.5 years), 81 nonpatient adolescents (56.8% males, mean age 15.5 ± 2.3 years), 87 adolescent psychiatric inpatients with no suicidal attempts (57.5% males, mean age 16 ± 3 years)	Ego Defense Scale (EDS) of the Child Suicide Potential Scales (Pfeffer et al. 1979) and LSI	Spectrum of Suicidal Behavior Scale of the Child Suicide Potential Scales (Pfeffer et al. 1979)	Suicidal behavior was positively correlated with LSI denial, displacement, and repression (*p* < 0.01), EDS regression (*p* < 0.05), denial, projection, introjection, and repression (*p* < 0.01), and negatively with EDS sublimation (*p* < 0.05).
Bakhtiari et al. (2023)	Iran	50 psychiatric inpatients with bipolar disorder (50% men, mean age 32.9 ± 10.2 years)	Primitive defense mechanisms scale of the Borderline Personality Inventory (Leichsenring 1999)	Beck Scale for Suicide Ideation (Beck et al. 1979)	Suicidal ideation was positively associated with the primitive defense mechanisms scale (*p* = 0.034).
Borst and Noam (1993)	The Netherlands	139 girls (mean age 14.4 ± 0.75 years) admitted to a psychiatric hospital classified as 59% pre‐conformists and 41% conformists according to social‐cognitive development: 37.4% with and 62.6% without previous suicide attempts	Adolescent version of the Defense Mechanisms Inventory (DMI; Gleser and Ihilevich 1969)	Diagnostic Interview Schedule for Children (Costello et al. 1985)	Among pre‐conformist girls, those with suicide attempts scored higher on the turning‐against‐self defense cluster than non‐suicidal girls (*p* = 0.005).
Brody and Carson (2012)	United Kingdom	114 nonclinical adolescents (43% males, mean age 17.1 ± 1.1 years)	40‐item version of the Defense Style Questionnaire (DSQ‐40; Andrews et al. 1993)	The Deliberate Self‐Harm item of the Youth Risk Behavior Survey (Brener et al. 1995)	Deliberate self‐harm was associated with a higher immature defenses score (OR = 6.3, 95% CI = 2.8‐14.3, Wald statistic = 19.1) and a lower mature defenses score (OR = 0.24, 95% CI = 0.12‐0.48, Wald statistic = 16.2).
Corruble et al. (2003)	France	77 inpatients (39% men, mean age 39.2 ± 11.5 years) with major depressive disorder	DSQ‐40	Lifetime number of suicide attempts according to the Schedule for Affective Disorders and Schizophrenia (Endicott 1978)	Number of suicide attempts was associated with neurotic defenses (pseudo‐altruism *p* = 0.03; undoing *p* = 0.05) and immature defenses (splitting *p* = 0.02; passive aggression *p* = 0.001; somatization *p* = 0.002; acting out *p* = 0.01; projection *p* = 0.0001).
Corruble et al. (2004)	France	156 depressed inpatients: 38.5% (33% men, mean age 39.9 ± 11.9 years) with and 61.5% (39% men, mean age 41.4 ± 12.4 years) without recent suicide attempt	DSQ‐40	Schedule for Affective Disorders and Schizophrenia (Endicott 1978)	Suicide attempters scored higher on the immature defense cluster (*p* = 0.006), especially on autistic fantasy (*p* = 0.007), passive aggression (*p* = 0.005), acting out (*p* = 0.001), and projection (*p* = 0.008).
Dedic et al. (2010)	Serbia	30 hospitalized patients following suicide attempts (20% men, mean age 28.2 ± 12.46 years) and 30 patients asking for psychiatric examination without suicide attempts (20% men, mean age 30 ± 12.78 years)	DSQ‐40	Suicide attempts by self‐poisoning (93% with tablets, 7% with corrosive agents)	Suicide attempters scored higher on the altruism neurotic defense (*p* = 0.05) and on the following immature defenses: dissociation (*p* = 0.05), projection, acting out, and devaluation (*p* = 0.01).
Dedic et al. (2019)	Serbia	90 patients on psychotherapeutic treatment (78.9% women, mean age 37.92 ± 11.04 years): 50 with and 40 without suicide attempts	DSQ‐40	Suicide attempt by self‐poisoning or self‐reported lifetime suicidal attempts during an outpatient psychiatric examination	Suicide attempters used more often immature defenses than controls (*p* = 0.001).
Fennig et al. (2005)	Israel	404 adolescents aged 12–21 years, hospitalized in two psychiatric units: 54% suicide attempters (34.7% males) and 46% without any history of suicide attempt (55.7% males)	EDS and LSI	Schedule for Affective Disorders and Schizophrenia for School‐age Children (Apter et al. 1989a)	Suicide attempters scored higher than non‐attempters on the LSI regression and displacement (*p* < 0.05), while on the EDS attempters scored lower on regression (*p* < 0.001) and undoing (*p* < 0.05).
Foto‐Özdemir et al. (2016)	Turkey	64 adolescents (39.1% males, mean age 14.8 ± 1.4 years) with a suicide attempt in the last 15 days	DMI	Admission to an emergency service because of a suicide attempt that occurred in the last 15 days	Adolescents with recurrent suicidal thoughts before the suicide attempt had significantly higher levels of turning‐against‐self than adolescents without a recurrent suicidal thought (*p* = 0.05).
Greenwald et al. (1994)	USA	74 male volunteers with alcohol dependence (mean age 38.6 years)	LSI	Suicide Risk Scale (Plutchik et al. 1989)	Higher regression (*p* ≤ 0.001) and displacement (*p* ≤ 0.01) scores were significantly associated with suicide risk.
Güzel et al. (2022)	Turkey	101 patients who had bariatric surgery (54.5% men, mean age 52.46 ± 9.72 years)	DSQ‐40	Suicide Probability Scale (Cull and Gill 1988)	The neurotic defenses score was associated with higher suicide risk (*p* = 0.003).
Hovanesian et al. (2009)	USA	75 patients with major depressive disorder (62.7% women): 35% with no suicide ideation/attempt (mean age 37.6 ± 11.4 years), 33% with suicide ideation but no attempt (mean age 41.9 ± 14.2 years), 32% with suicide attempt (mean age 37.5 ± 11.7 years)	DSQ‐40	Clinical interview completed within 48 h since admission to an emergency room or a psychiatric unit	Greater use of image‐distorting defense mechanisms predicted the membership in the suicide attempt group (*p* < 0.05).
Ishfaq and Kamal (2024)	Pakistan	509 imprisoned men (mean age 35 ± 10.21 years)	DSQ‐40	The suicidal ideation item of the Cross‐Cutting Symptom Measure (American Psychiatric Association 2013)	A high suicidal ideation score was associated with lower scores on mature and neurotic defenses (*p* < 0.01) and with higher scores on immature defenses (*p* < 0.05).
Lee et al. (2020)	Republic of Korea	125 borderline personality disorder patients (43.7% men, mean age 28.39 ± 9.18 years): 33.6% with and 66.4% without a history of one or more suicide attempts	78‐item version of the Defense Style Questionnaire (Bond 1986)	Preliminary data sheets assessed the number and methods of suicide attempts	Suicide attempters scored higher on splitting of other's image (*p* = 0.021), projective identification (*p* = 0.005), and affiliation (*p* = 0.003) than non‐suicidal patients.
Lee et al. (2022)	Republic of Korea	258 psychiatric patients (54% women, mean age 29.8 ± 13.3 years): 50% with suicidal behavior (ideation or attempts) and 50% without suicidal behavior	Ewha Defense Mechanisms Test (Kim et al. 1991)	Emergency Department visit with one of the patients' chief complaints being suicidal ideation or attempt	Among patients with suicidal behavior, those with suicidal attempts scored higher on rationalization than patients with suicidal ideation (*p* = 0.03).
Moradinazar et al. (2019)	Iran	200 patients with suicide attempts by self‐poisoning (77% men, mean age 28.1 ± 7 years) and 200 non‐suicidal patients hospitalized for nondeliberate poisoning (80.5% men, mean age 28 ± 1 years)	DSQ‐40	Hospitalization because of deliberate self‐poisoning	Compared to non‐suicidal patients, suicide attempters scored higher on immature defenses (devaluation *p* = 0.01; acting out *p* = 0.002; somatization *p* = 0.01; fantasy *p* = 0.006; passive aggressive behavior *p* = 0.02; compartmentalization *p* = 0.03) and neurotic defenses (pseudo‐altruism *p* < 0.001; undoing *p* = 0.01), and lower on mature defenses (suppression *p* < 0.001; humor *p* = 0.01; anticipation *p* = 0.01).
Mozafari et al. (2024)	Iran	384 (39.8% adolescents and 60.2% adults) outpatients with suicidal ideation and mental disorders (43.5% men, mean age 27.84 ± 3.56 years for women and 32.16 ± 4.83 years for men)	DSQ‐40	Beck Scale for Suicidal Ideation (Beck et al. 1979)	In structural equation modeling, immature and neurotic defense mechanisms had positive relationships with suicidal ideation, while mature defense mechanisms had negative association with suicidal ideation.
Peng et al. (2023)	China	64 major depressive disorder patients with non‐suicidal self‐injury (73.4% women, mean age 22.47 ± 3.63 years), 60 major depressive disorder patients without non‐suicidal self‐injury (71.7% women, mean age 22.53 ± 3.86 years), and 64 healthy control subjects (71.9% women, mean age 22.83 ± 3.97 years)	88‐item version of the Defense Style Questionnaire (Andrews 1989)	DSM‐5 diagnostic criteria for non‐suicidal self‐injury (American Psychiatric Association 2013) Ottawa Self‐Injury Scale (Nixon et al. 2015)	Depressed patients with non‐suicidal self‐injury scored higher on the immature defense style than depressed patients without non‐suicidal self‐injury (*p* = 0.001) and higher on the intermediate defense style than healthy controls (*p* = 0.034).
Peng et al. (2024)	China	80 major depressive disorder patients with non‐suicidal self‐injury and suicidal attempts in the last year (86.25% women, mean age 20.20 ± 4.75 years) and 50 patients with major depressive disorder and non‐suicidal self‐injury without suicidal attempts (94% women, mean age 22.32 ± 4.51 years)	88‐item version of the Defense Style Questionnaire (Andrews 1989)	Ottawa Self‐Injury Scale (Nixon et al. 2015)	No significant differences were found in immature, intermediate and mature defense styles between the two groups.
Pfeffer et al. (1982)	USA	65 child psychiatric inpatients (73.8% males, mean age 10.1 years): 21.5% with no suicidal ideas or acts, 26.2% with suicidal ideation, and 52.3% with suicidal acts	EDS	Spectrum of Suicidal Behavior Scale of the Child Suicide Potential Scales (Pfeffer et al. 1979)	Introjection (*p* < 0.01), displacement (*p* < 0.05), and the total defense score (*p* < 0.05) were positively associated with suicidal behavior.
Pfeffer et al. (1984)	USA	101 school children (70.3% males, mean age 9.7 ± 1.2 years)	EDS	Spectrum of Suicidal Behavior Scale of the Child Suicide Potential Scales (Pfeffer et al. 1979)	Introjection was higher in school children with suicidal thoughts or acts compared to those without evidence of suicidal tendencies (*p* = 0.004).
Pfeffer et al. (1986)	USA	106 child psychiatric inpatients (76.4% males, mean age 10.9 ± 1.3 years), 101 child psychiatric outpatients (67.3% males, mean age 9.4 ± 1.2 years), 101 child non‐patients (70.3% males, mean age 9.7 ± 1.2 years)	EDS	Spectrum of Suicidal Behavior Scale of the Child Suicide Potential Scales (Pfeffer et al. 1979)	Among the child psychiatric inpatients, total defense score, introjection, and displacement were associated with suicidal behavior (*p* < 0.05).
Pfeffer et al. (1988)	USA	75 school children (69.3% males, mean age 12.1 ± 0.25 years)	EDS	Spectrum of Suicidal Behavior Scale of the Child Suicide Potential Scales (Pfeffer et al. 1979)	Denial (*p* = 0.03), reaction formation (*p* = 0.03), and projection (*p* = 0.05) were associated with suicidal behavior.
Pfeffer et al. (1995)	USA	133 children (72.9% males, mean age 10.5 ± 1.8 years, 19% suicide attempters, 21% with suicide ideation, 12% non‐suicidal psychiatric patients, and 48% nonpatients) reinterviewed 6‐8 years later (mean age 17 ± 2.2 years)	EDS	Spectrum of Suicidal Behavior Scale of the Child Suicide Potential Scales (Pfeffer et al. 1979)	Suicide attempts were associated with projection (*p* = 0.004), regression (*p* = 0.002), compensation (*p* = 0.009), and reaction formation (*p* = 0.004). Suicidal ideation was associated with projection (*p* = 0.0002), reaction formation (*p* = 0.01), and compensation (*p* = 0.024).
Recklitis et al. (1992)	USA	200 adolescent psychiatric patients (42% males, mean age 14.1 ± 1.02 years): 34% suicide attempters, 13.5% with suicide ideation, and 52.5% non‐suicidal patients	DMI	Diagnostic Interview Schedule for Children (Costello et al. 1985)	Adolescents with suicide attempts and those with suicidal ideation scored higher on turning‐against‐self than non‐suicidal patients (*p* < 0.01). Adolescents with suicide attempts scored lower on reversal than non‐suicidal patients (*p* < 0.05).
Sarno et al. (2010)	Italy	578 University students (82.5% women, mean age 22.3 ± 3.4 years): 20.6% of them with at least one lifetime episode of self‐injury	Response Evaluation Measure‐71 (Steiner et al. 2001)	Deliberate Self Harm Inventory (Gratz 2001)	Recurrent self‐injurers (≥ 5 episodes of self‐injury) scored higher than subjects without self‐injury episodes in projection, dissociation, undoing, repression, fantasy, and withdrawal (*p* ≤ 0.001).
Scholz (1973)	USA	35 suicide attempters and 35 matched non‐suicidal neuropsychiatric controls (65.7% women, age range 18–50 years)	DMI	Q‐sort ratings based on Leonard (1967) guidelines for recognition of suicidal types	Suicidal patients scored higher on turning‐against‐self than controls (*p* < 0.002).
Xia et al. (2023)	China	31 adolescent patients with non‐suicidal self‐injury (58.1% males, mean age 17.71 ± 2.04 years) and 60 matched controls without non‐suicidal self‐injury (60% males, mean age 17.58 ± 2.1 years)	88‐item version of the Defense Style Questionnaire (Andrews 1989)	DSM‐5 diagnostic criteria for non‐suicidal self‐injury (American Psychiatric Association 2013)	Compared to controls, adolescents with non‐suicidal self‐injury scored higher in immature defenses (projection *p* = 0.015; passive aggression *p* < 0.001; subliminal manifestation *p* < 0.001; somatization *p* < 0.001), and lower in mature defenses (sublimation *p* < 0.001; humor *p* = 0.006).

These defenses all involve varying degrees of reality distortion and different approaches to managing stress. They are organized around two fundamental mechanisms: repression and splitting. In the defenses organized around repression (removal‐based or neurotic), the primary goal is to remove conflicting themes from awareness. Conversely, splitting‐based defenses divide conflicting and emotionally intense perceptions to avoid internal conflict (Kernberg 1984). All these defense mechanisms contribute to creating internal conditions of vulnerability to self‐injurious behaviors. Immature defenses, such as splitting, projection, and projective identification, distort the subjective perception of the self and the others, leading to misunderstandings in communication. Similarly, acting out allows individuals to face conflicts through impulsive actions, with apparent disregard for social consequences. In both cases the consequence is the loss of social support. A crucial aspect of suicidal experience is the pervasive sense of isolation and lack of perceived resources, combined with an impaired ability to seek and accept help or maintain emotionally supportive relationships (Motillon‐Toudic et al. 2022).

Dissociation disrupts the integrative capacities of identity, leading to inadequate self‐esteem along with quick mood fluctuations. If the individual fails to establish a stable and consistent sense of identity, identity confusion and problems in self‐integration arise and the outcome includes a greater risk for self‐injurious behaviors (Sekowski et al. 2024).

Some of the included studies found a significant role of turning‐against‐self strategies in self‐injurious behaviors. Theoretical formulations propose that the suicidal individual is unable to tolerate feelings of anger and hostility against significant others. He or she defends against these feelings by redirecting them inward toward the self. In response to conflictual situations suicidal subjects are more likely to find fault with themselves and their actions (Kernberg 1984). In this regard, in a longitudinal study using machine learning techniques, an internalizing personological functioning, together with interpersonal vulnerability, were the strongest predictors of suicide attempts in borderline personality disorder patients (Fortaner‐Uyà et al. 2025).

## 4 Discussion

The objective of this study was to systematically review and synthesize existing quantitative research examining the association between defense mechanisms and self‐injurious behaviors, including suicidal ideation, suicide attempts, and NSSI, with the aim of clarifying the role of intrapsychic defensive functioning in the development and maintenance of self‐destructive behaviors across clinical and non‐clinical populations.

To our knowledge, this is the first systematic review addressing the relationship between defenses and self‐injurious behaviors. Drawing on data from 30 quantitative studies retrieved from PubMed, Web of Science, PsycInfo, and Scopus databases, the present review identified a consistent association between immature and neurotic defense styles and increased risk for self‐injurious behaviors across various populations, including adolescents, individuals with psychiatric disorders, incarcerated individuals, persons with alcohol dependence, and post‐bariatric surgery patients.

The findings reveal that immature and neurotic defenses, such as splitting, projection, acting out, and dissociation, are consistently linked to higher levels of self‐injurious behaviors. These defenses are typically associated with distorted self‐perception, impaired emotional regulation, and diminished social support, all of which heighten vulnerability to self‐harm (Lee et al. 2022; Mozafari et al. 2024). In contrast, mature defenses appear less frequently among individuals who engage in self‐injurious behaviors (Xia et al. 2023). However, this difference tends to be less pronounced in non‐clinical samples (Brody and Carson 2012; Sarno et al. 2010). It is worth noting that NSSI and suicidal behavior may lie on a continuum of severity, although defense mechanisms remain relevant even when NSSI is conceptualized as distinct from suicidality (Fox et al. 2015).

Several explanations may account for the observed findings. First, the results of the present review reflect a convergence of psychological theory, clinical observation, and empirical data. Defense mechanisms are core components of personality functioning, particularly in managing conflicts, stress, and trauma. Psychodynamic theories posit that immature defenses distort reality and impair relationships, thereby exacerbating internal distress and reducing the individual's capacity to cope (Di Giuseppe et al. 2019; Kernberg 1984). Empirical studies support this view, suggesting that individuals under high stress or with a history of trauma may default to less adaptive defenses, thereby increasing their risk of engaging in self‐injurious behaviors (Bakhtiari et al. 2023; Dedic et al. 2019; Lee et al. 2020; Moradinazar et al. 2019; Mozafari et al. 2024).

Second, it is plausible to conceptualize defense mechanisms as mediators linking personality traits and psychopathology (Peng et al. 2023, 2024). The review frames defenses as a bridge between personality traits (e.g., impulsivity, identity diffusion) and mental health outcomes, such as depression or suicidality. This conceptual framework offers insights into why certain individuals may respond to psychological distress with self‐injurious behaviors (Corruble et al. 2003; Mozafari et al. 2024).

Equally important is the empirical consistency of the findings across diverse populations. Despite differences in sample characteristics (e.g., adolescents, patients with psychiatric disorders, incarcerated individuals), studies consistently found that immature/neurotic defenses are more common in individuals who engage in self‐harm (Dedic et al. 2010; Greenwald et al. 1994; Güzel et al. 2022; Ishfaq and Kamal 2024). This consistency suggests that the maturity level of defense mechanisms may serve as a transdiagnostic risk factor.

Supporting this interpretation is the evidence linking emotional dysregulation and social support deficits to immature defense usage. Immature defenses are associated with heightened emotional reactivity, increased interpersonal conflict, and difficulty seeking or maintaining social support ‐‐ all well‐established risk factors for self‐injurious behaviors. These mechanisms perpetuate feelings of isolation, low self‐worth, and hopelessness, reinforcing a cycle of self‐destructive tendencies (Lee et al. 2022; Motillon‐Toudic et al. 2022; Mozafari et al. 2024; Sekowski et al. 2024).

### 4.1 Limitations of the Studies

Despite the intriguing insights, a comprehensive analysis of the literature to date reveals certain limitations. A point of reflection leads us to focus on how defense mechanisms have been operationalized and investigated in the studies under consideration. Many researchers underscore that the defining characteristic of defense mechanisms is their unconscious nature (Cramer 2015), which poses significant methodological challenges. Specifically, if defense mechanisms function largely outside of conscious awareness, how can individuals reliably self‐report them? Most studies included in this review relied on self‐report measures, due to their ease of use, cost‐effectiveness, and suitability for large‐scale administration. It is essential to highlight that this approach is inherently characterized by potentially significant methodological limitations. However, some authors emphasize that such an evaluative methodology still provides an opportunity to investigate the conscious derivatives of unconscious defenses—observable behavioral patterns that indirectly reflect an individual's defensive style (Bond 2004). These conscious manifestations offer valuable insights into an individual's intrapsychic functioning. This perspective allows the studies included in the present review to be regarded as a valuable contribution to our understanding of the association between defenses and self‐injurious behaviors.

A second methodological issue concerns the classification of defenses. Is it possible to generate a standardized yet clinically relevant taxonomy of defense mechanisms that is neither overly reductive nor excessively complex? The absence of consensus on this issue complicates both data collection and interpretation.

Furthermore, there is a lack of studies on the role of defenses in suicidality in medical populations, such as individuals with cancer or chronic pain, where the issue of self‐injurious behaviors is particularly relevant. To date, only one study has reported a consistent association between immature defenses and self‐injurious behaviors in patients following bariatric surgery (Güzel et al. 2022).

Developmental and contextual factors should also be considered. For example, immature defenses are developmentally common in adolescence but become more problematic when combined with psychopathology, contributing to an elevated risk for self‐harm behavior. It would be valuable to conduct more studies in child and adolescent populations to enable the early identification of individuals who are developing dysfunctional defensive mechanisms, increasing the risk for suicidality as they grow older. Some existing studies involving children have reported associations between specific defenses such as projection, introjection, regression, compensation, and reaction formation and suicide thoughts or acts (Pfeffer et al. 1995). Further studies are needed to confirm and replicate these findings.

Elderly populations are also underrepresented, despite being particularly vulnerable due to unique stressors (e.g., chronic illness, loss, existential anxiety). There is a shortage of studies focusing on the elderly, which is an age group at increased risk for suicidal behavior (Beristianos et al. 2016). Examining defensive structures in this population may reveal how depression and despair, caused by the inevitable awareness of aging and the progressive loss of opportunities, contribute to an increased risk for self‐injurious behavior. Moreover, environmental stressors such as trauma, social exclusion, and chronic pain interact with defense mechanisms to influence behavioral outcomes.

A final limitation pertains to the predominantly cross‐sectional design of the studies, and the absence of longitudinal studies which precludes the examination of changes in self‐injurious behaviors over time and limits causal inference. While cross‐sectional findings suggest that immature defenses increase the risk for self‐injurious behaviors, the directionality of this relationship remains unclear: immature defenses may contribute to self‐harm, or conversely, self‐harming behaviors may further establish immature defenses.

In summary, several methodological constraints must be acknowledged, including measurement issues, sample diversity, and study design.

### 4.2 Limitations and Implications of the Review

This review has different limitations. First, we did not perform meta‐analysis and thus our conclusions can only be qualitative in nature. Second, the results of the review are updated to January 2025, therefore further updates could eventually find data different from the present results. There are also some limitations regarding the search strategy. In the electronic searches we only include the general term “defense mechanism*.” The high number of individual defenses described in literature has hindered their inclusion as keywords. However, we cannot exclude that the use of individual defense mechanisms as keywords would have resulted in a more accurate search strategy. Consistently with this limitation, six out of 30 articles were retrieved by means of reference searching. Thus, it cannot be excluded that other terms would have been relevant to the search strategy. Another limitation of the review was the lack of a third author for arbitration in case of initial disagreement about whether to include an article in the review or not.

Despite these limitations, the present systematic review highlights how defensive patterns and their level of maturity could be helpful in reducing risk factors for self‐injurious behaviors. These findings provide incremental information for the improvement of preventive and therapeutic strategies aimed at reducing dangerous behaviors that threaten survival in both clinical and non‐clinical populations.

Predicting a suicide attempt or a self‐injurious behavior remains a challenging objective. However, it is of considerable clinical interest to consider the role of defensive functioning in self‐injurious behaviors. Defense mechanisms have predictive significance in guiding the diagnostic assessment and constitute a critical target for early interventions and the facilitation of psychological change (Conversano et al. 2023). A growing body of research has examined how defense mechanisms change throughout the course of therapy and how they become more adaptive over the course of psychological treatments across various patient populations (de Roten et al. 2021; Perry and Bond 2012).

These findings highlight the value of integrating therapeutic efforts with defense mechanisms to prevent self‐injurious behaviors. Adjusting defenses configuration may modulate the levels of emotion produced by stress, keep impulsiveness under control, and help an individual dealing with life traumas and losses.

Defenses, therefore, represent an area where early intervention could be implemented as part of a preventive strategy to reduce both non‐suicidal self‐injury behavior and suicide risk. Routine evaluation of defensive functioning should thus be methodically included in the psychological assessment of patients with self‐harming ideation or behaviors, going beyond the severity of associated symptomatology. This topic is particularly relevant when addressing clinical interventions in adolescent populations, where defenses are still in the process of consolidation and remain subject to remodeling and therapeutic adjustment.

## 5 Conclusions

The results of the present systematic review are supported by a strong theoretical framework, consistent empirical evidence, and plausible psychological mechanisms linking immature defenses to self‐injurious behaviors. While methodological limitations exist, particularly in measuring unconscious processes, the evidence provides a convincing rationale for incorporating defense mechanisms assessment into risk evaluations, preventive efforts, and treatment planning for individuals at risk for self‐harming behavior. However, future research would benefit by employing longitudinal designs, by including underrepresented populations (e.g., older adults, medically ill individuals), and by utilizing multi‐method assessment strategies to deepen our understanding of this complex relationship.

## Funding

The authors received no specific funding for this work.

## Conflicts of Interest

The authors declare no conflicts of interest.

## Supporting information


**Supplementary Table 1:** Quality assessment of the included articles according to the National Heart, Lung, and Blood Institute tool for observational cohort and cross‐sectional studies.

## Data Availability

The data that support the findings of this study will be available from the moment of publication in AMS Acta Institutional Research Repository at https://doi.org/10.6092/unibo/amsacta/8634, URI https://amsacta.unibo.it/id/eprint/8634.
